# Experimental and machine learning approaches to investigate the effect of waste glass powder on the flexural strength of cement mortar

**DOI:** 10.1371/journal.pone.0280761

**Published:** 2023-01-23

**Authors:** Muhammad Nasir Amin, Hassan Ali Alkadhim, Waqas Ahmad, Kaffayatullah Khan, Hisham Alabduljabbar, Abdullah Mohamed

**Affiliations:** 1 Department of Civil and Environmental Engineering, College of Engineering, King Faisal University, Al-Ahsa, Saudi Arabia; 2 Department of Civil Engineering, COMSATS University Islamabad, Abbottabad, Pakistan; 3 Department of Civil Engineering, College of Engineering in Al-Kharj, Prince Sattam Bin Abdulaziz University, Al-Kharj, Saudi Arabia; 4 Research Centre, Future University in Egypt, New Cairo, Egypt; Instituto Superior Tecnico, Universidade de Lisboa, Erbil Polytechnic University, PORTUGAL

## Abstract

Using solid waste in building materials is an efficient approach to achieving sustainability goals. Also, the application of modern methods like artificial intelligence is gaining attention. In this regard, the flexural strength (FS) of cementitious composites (CCs) incorporating waste glass powder (WGP) was evaluated via both experimental and machine learning (ML) methods. WGP was utilized to partially substitute cement and fine aggregate separately at replacement levels of 0%, 2.5%, 5%, 7.5%, 10%, 12.5%, and 15%. At first, the FS of WGP-based CCs was determined experimentally. The generated data, which included six inputs, was then used to run ML techniques to forecast the FS. For FS estimation, two ML approaches were used, including a support vector machine and a bagging regressor. The effectiveness of ML models was assessed by the coefficient of determination (R^2^), k-fold techniques, statistical tests, and examining the variation amongst experimental and forecasted FS. The use of WGP improved the FS of CCs, as determined by the experimental results. The highest FS was obtained when 10% and 15% WGP was utilized as a cement and fine aggregate replacement, respectively. The modeling approaches’ results revealed that the support vector machine method had a fair level of accuracy, but the bagging regressor method had a greater level of accuracy in estimating the FS. Using ML strategies will benefit the building industry by expediting cost-effective and rapid solutions for analyzing material characteristics.

## 1. Introduction

Several processes, including manufacturing, electricity generation, mining, agricultural production, iron and steel metallurgy, and the creation of electronic devices, generate large amounts of waste [1,2]. Several hazardous waste materials are combustible, corrosive, infectious, incendiary, and chemically volatile, and their disposal in landfills has resulted in substantial financial losses [2–4]. Therefore, it is desirable to recycle or reuse waste materials in building materials. Cementitious composites (CCs) are widely employed in construction [5–8]. Numerous methods have been used by researchers to enhance CC performance [9–11]. Waste materials may be used in place of natural aggregates [12–14], fibers for reinforcement [15–19], and cement [20–22] to improve the performance of CCs. Employing the methods, cement and aggregate use might be reduced, conserving natural resources, and reducing CO_2_ emissions [23–27]. The mechanical performance of CCs has been shown to improve with the addition of some waste materials [28,29]. As a result of its abundance, waste glass (WG) is commonly found in landfills across the world [30–32]. Particularly, large urban areas are facing a dramatic decline in landfill space despite their populations producing ever-greater quantities of municipal solid waste [33]. WG is chemically more robust than other common waste materials like wood and plastic. Buried WG does not biodegrade for a longer period [34]. Some glasses, such as cathode ray tubes (CRTs), comprise toxic components like lead, beryllium, mercury, and cadmium, infecting groundwater and soil [35]. Around 43 million tons of CRT glass are manufactured yearly in China [36], posing a serious threat to the environment and people’s health.

To manufacture glass, silica must be melted for hours at a high temperature [37]. The amount of energy required for this is rather high. The temperature is held at 1500°C for 24 hours for container glass and 72 hours for plate glass [38]. To produce 1 kg of plate glass uses around 17 J of energy from fossil fuels and discharges roughly 0.6 kg of CO_2_ [39]. Glass manufacturing consumes more than 350 PJ of energy per year in Europe, which is about 20% of the region’s total industrial energy consumption [39]. So, it is vital to pay close attention to how WG can be properly recycled. One frequent way that WG is reused is in the reprocessing of glass goods. But reprocessing is complicated since glass items are made from WG that have been cleaned, separated, and melted [40]. Another way to recycle WG is to use it in the production of building materials. Crushed WG might be utilized in CCs as a partial cement and aggregate replacement [41,42]. There are a number of benefits associated with using WG in CCs. First, WG doesn’t require melting; therefore, less energy is consumed. The second perk of WG is how simple it is to handle; specifically, there is no need to sift and clean glass. Third, there will be a higher need for WG due to the widespread use of CCs in the building. Glass’s hazardous components can be encased and rendered inert by CCs. WG recycling in CCs has been shown to be the best approach in earlier research [43,44]. Conservation of natural raw materials and simplified waste management are additional benefits of using WG as an aggregate substitute in CCs. However, WG utilization as cement replacement will decrease cement requirements and CO_2_ discharges [45,46].

Flexural strength (FS) is an important factor to consider when designing concrete structures, as it influences flexural cracking, deflection characteristics, brittleness ratio, and shear strength [47]. The selection of CC’s constituents and the prediction of its output engineering property, such as FS, is a common challenge. This is due to the fact that CC is a heterogeneous mixture consisting of several elements [48]. To reduce the expense of doing more tests, it is crucial to create strong and trustworthy prediction models based on the current input and output data [49–51]. Appropriate prediction models also permit reductions in the number of trivial input combination searches that may result in desired tangible performance [52]. Consequently, they permit substantial cost and time savings. Creating such models is difficult due of the very nonlinear relationship between the input elements and the output concrete strengths. In the previous few decades, major attempts have been made to apply intelligent computing algorithms to civil engineering challenges [53]. Predicting material properties has been done using data-driven methods [54–56]. In order to estimate material characteristics, researchers have developed predictive models with the ultimate objective of minimizing prediction error relative to experimental data [57,58]. Artificial intelligence strategies, like machine learning (ML), are amongst the most innovative modeling strategies for the said purpose. Most of the former ML-related publications concentrated on foretelling the strength of normal CCs [59,60], whereas just a few concentrated on predicting the features of CCs with WG.

This research employed experimental and ML strategies to estimate the FS of CCs incorporating waste glass powder (WGP). The WG was gathered from local construction detritus, cleansed, and ground into a powder. The CC samples were cast with different concentrations of WGP as a fine aggregate and cement replacement (0–15%). The FS of WGP-based CC was determined using experimental approaches at 28 days of age. Following the completion of the experiments, the obtained data were utilized to develop ML prediction models. Support vector machine (SVM) and bagging regressor (BR) were used to accomplish the research’s objectives. SVM is a single ML technique, whereas BR is an ensemble ML technique [61]. Each model’s performance was calculated using the coefficient of determination (R^2^), the k-fold approach, statistical tests, and the variance in projected outcomes (errors). This research is new in that it evaluated the FS of CCs incorporating WGP using experimental and ML approaches, involving both single and ensemble ML approaches. Material collecting, sample casting, curing, and conducting tests require a great deal of time, money, and effort in experimentally-based investigations. The building industry will profit from the elimination of these difficulties by utilizing innovative approaches like ML. Therefore, this research aimed to increase understanding regarding the application of ML approaches to predict material attributes. The obtained data from the experimental program may be utilized to train ML systems and assess material characteristics. This work employed six input parameters and 117 specimens to predict the FS of CCs incorporating WGP and evaluate the effectiveness of each ML technique.

## 2. Methods

### 2.1. Experimental strategy

**Fig 1:**
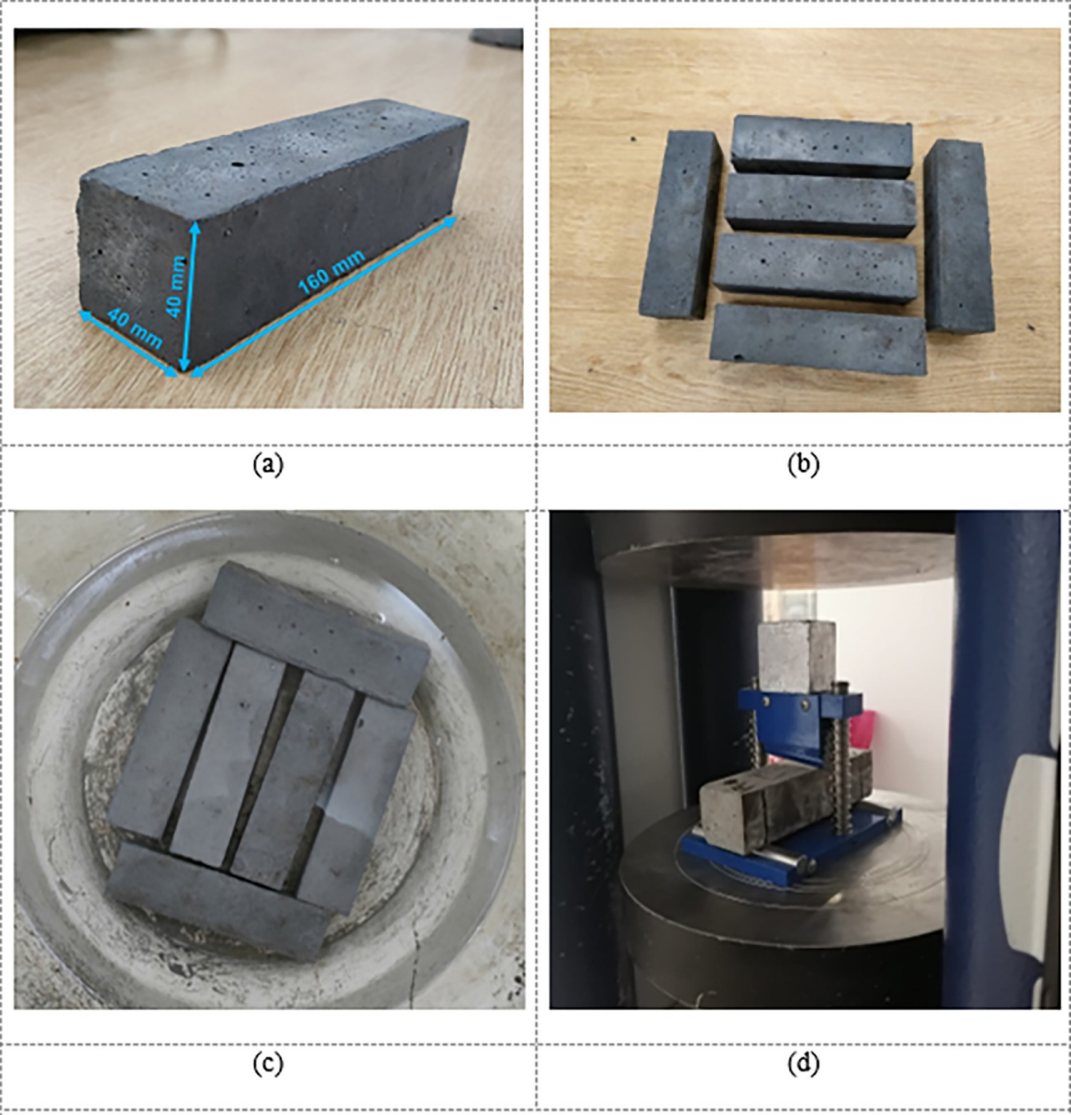
Images of the experimental program: (a) Dimensions of a specimen; (b) Specimens after demolding; (c) Water curing of specimens; (d) Testing assembly.

Locally accessible fine aggregate and Portland cement were gathered, whilst superplasticizer and silica fume were bought from Pakistan’s PAGEL Chemicals. The fineness modulus of fine aggregate was 2.6, bulk density was 1230 kg/m^3^, and specific gravity was 2.65. WG was gathered from nearby construction waste, cleaned, and mechanically ground into powder in the lab, and then sieved through a number 200 mesh. Three kinds of mix designs were selected for CCs, and their details are provided in Table 1. In all the mixes, the ratio of cement to sand was kept at 1:1, the water to cement ratio was 0.25, superplasticizer dosage at 4% by mass of cement. The only difference in the mixes was the silica fume (SF) content. In Mixes-1, 2, and 3, the SF content was 15%, 20%, and 25% by mass of cement. Additionally, WGP was substituted for cement and fine aggregate in all mixes in amounts from 0 to 15% at 2.5% increments. All the components of CCs were blended using a mechanical mixer. The mixer pan was filled with cement, silica fume, fine aggregate, and WGP. Half of the water containing the superplasticizer was then added, and the mixer was spun for two minutes. The mixer was turned for a further two minutes before the second half of the water was incorporated in two increments into the mixing bowl. Four minutes were used for mixing in total. To test the FS, specimens of 40 mm x 40 mm x 160 mm were cast. For each formulation, a set of three samples was cast; altogether, 117 samples were cast and examined. The average of three specimens tested for each formulation was used as the FS. Following casting, the specimens were held in models for 24 hours at room temperature before being demolded and stored in water for curing. Before testing, all the samples were cured for 28 days. The three-point load test required by ASTM C348-21 [62] was used for the FS test, with a loading rate of 0.1 mm/min in displacement-controlled mode. Fig 1 displays pictures of the test apparatus and specimens.

**Table 1:** Detail of mix proportion used during the experimental work.

Mix ID	Material substituted	Percentage substituted (%)	Cement (kg/m^3^)	Fine aggregate (kg/m^3^)	Silica fume (kg/m^3^)	Water (kg/m^3^)	Superplasticizer (kg/m^3^)	WGP (kg/m^3^)
Mix-1	-	-	810	810	122	203	40.5	0
	Cement	2.5	789.75	810	122	203	40.5	20.25
		5.0	769.5	810	122	203	40.5	40.5
		7.5	749.25	810	122	203	40.5	60.75
		10.0	729	810	122	203	40.5	81
		12.5	708.75	810	122	203	40.5	101.25
		15.0	688.5	810	122	203	40.5	121.5
	Sand	2.5	810	789.75	122	203	40.5	20.25
		5.0	810	769.5	122	203	40.5	40.5
		7.5	810	749.25	122	203	40.5	60.75
		10.0	810	729	122	203	40.5	81
		12.5	810	708.75	122	203	40.5	101.25
		15.0	810	688.5	122	203	40.5	121.5
Mix-2	-	-	760	760	153	191	38	0
	Cement	2.5	741	760	153	191	38	19
		5.0	722	760	153	191	38	38
		7.5	703	760	153	191	38	57
		10.0	684	760	153	191	38	76
		12.5	665	760	153	191	38	95
		15.0	646	760	153	191	38	114
	Sand	2.5	760	741	153	191	38	19
		5.0	760	722	153	191	38	38
		7.5	760	703	153	191	38	57
		10.0	760	684	153	191	38	76
		12.5	760	665	153	191	38	95
		15.0	760	646	153	191	38	114
Mix-3	-	-	720	720	180	180	36	0
	Cement	2.5	702	720	180	180	36	18
		5.0	684	720	180	180	36	36
		7.5	666	720	180	180	36	54
		10.0	648	720	180	180	36	72
		12.5	630	720	180	180	36	90
		15.0	612	720	180	180	36	108
	Sand	2.5	720	702	180	180	36	18
		5.0	720	684	180	180	36	36
		7.5	720	666	180	180	36	54
		10.0	720	648	180	180	36	72
		12.5	720	630	180	180	36	90
		15.0	720	612	180	180	36	108

### 2.2. Modeling strategy

**Fig 2:**
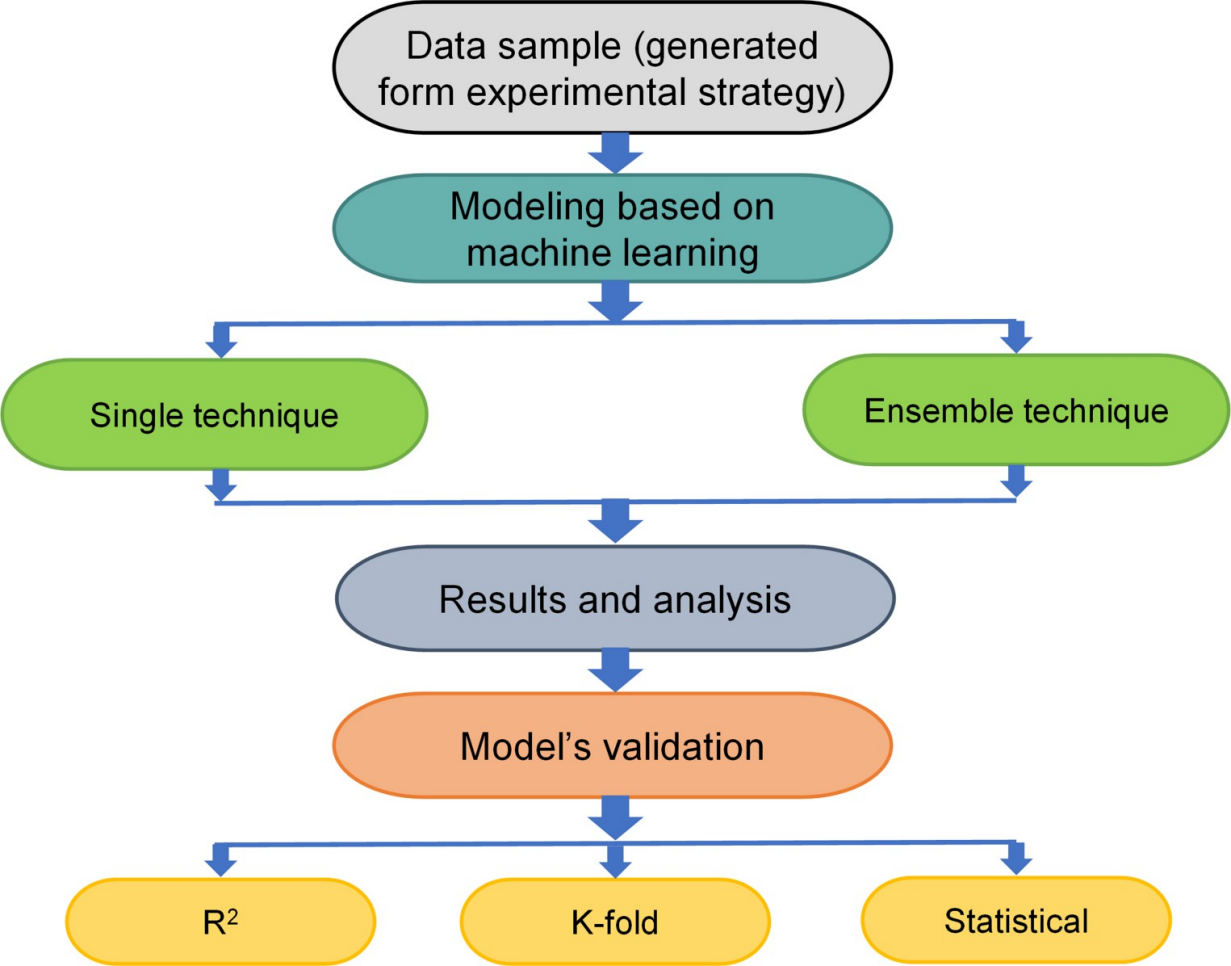
Sequence of ML and validation strategies adopted.

ML approaches require a comprehensive range of input parameters to build the anticipated output [63]. With the help of the experimental findings, the FS of CCs containing WGP was calculated. The techniques used cement, water, fine aggregate, WGP, superplasticizer, and silica fume as input features and FS as the output. Both single and ensemble ML algorithms were employed alongside Python code and the Spyder (version 5.1.5) from the Anaconda Navigator program. While BR was used as an ensemble ML approach, SVM was used as a single ML method. These ML techniques are frequently utilized to forecast desired outcomes using input parameters. These techniques might be used to estimate the strength of CCs, temperature effects, and durability [64,65]. For testing and training during the modeling phase, the allocation of experimental data was 30% and 70%, respectively. The R^2^ score of the predicted outcome indicates how well the models operated. The R^2^ value suggests the level of variance; a number near 0 denotes greater variation, whilst a value nearby 1 suggests that the experimental results and prediction model are almost perfectly suited [66]. Additionally, ML models were subjected to statistical, k-fold, and error analysis, like mean absolute error (MAE), root mean square error (RMSE), and mean absolute percentage error (MAPE). Fig 2 shows the flowchart of the modeling program. The ML approaches and validation strategies used in this work are described in the following sub-segments.

#### 2.2.1. Support vector machine

**Fig 3:**
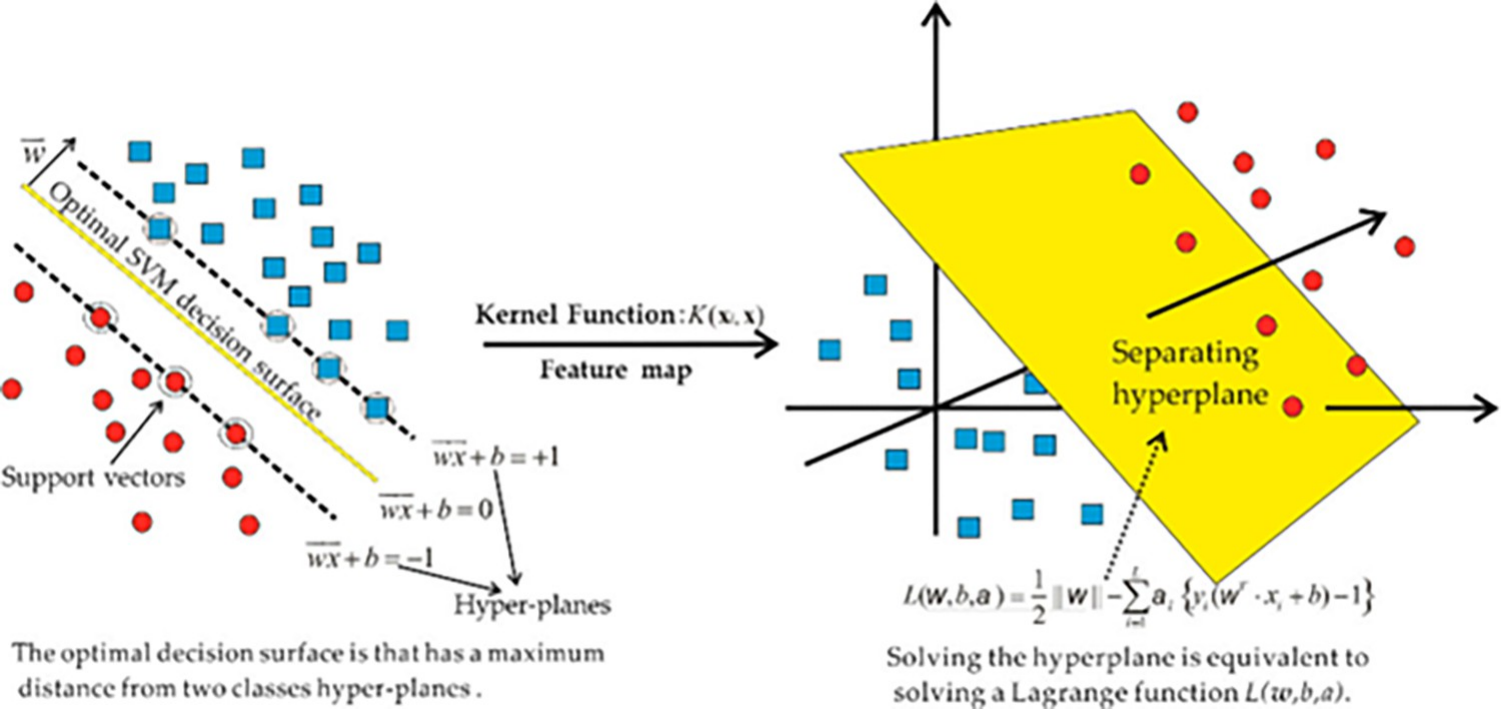
Map of SVM strategy [67].

**Fig 4:**
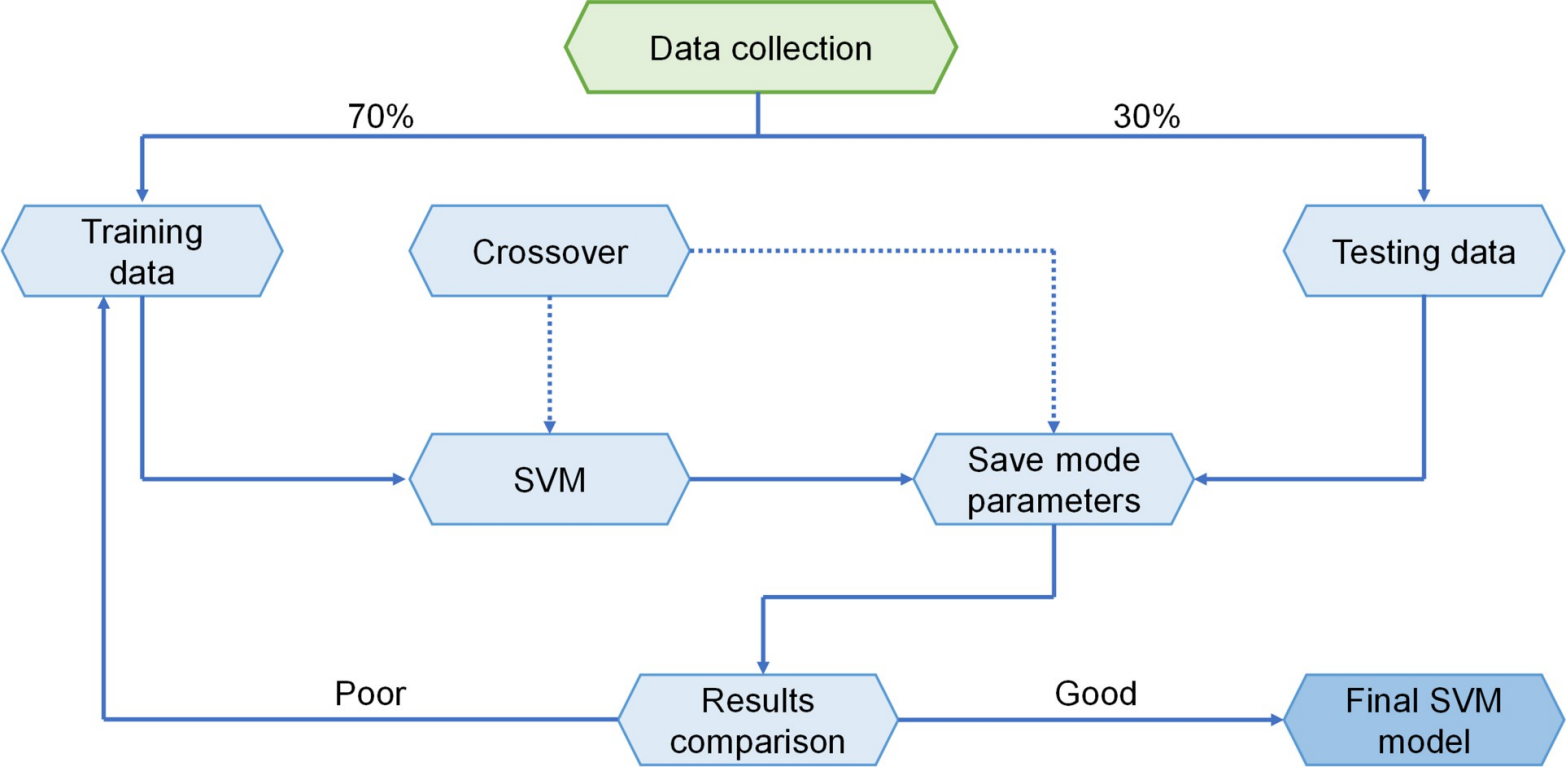
Sequence of SVM technique [68].

SVM is a subset of supervised ML algorithms that are used to assess data for classification and regression. A discrete vector, i.e., a line or plane with the biggest feasible gap, is used to differentiate the forms of the various classifications in an SVM technique, which represents the data as points in space. Fig 3 shows how new examples are categorized according to which side of the vector they are located. The SVM model’s implementation strategy is shown in Fig 4. The strength of the material is evaluated using this technique, which takes into consideration the impact of many components. The parameters of the SVM model are chosen using the optimization approach.

#### 2.2.2. Bagging regressor

**Fig 5:**
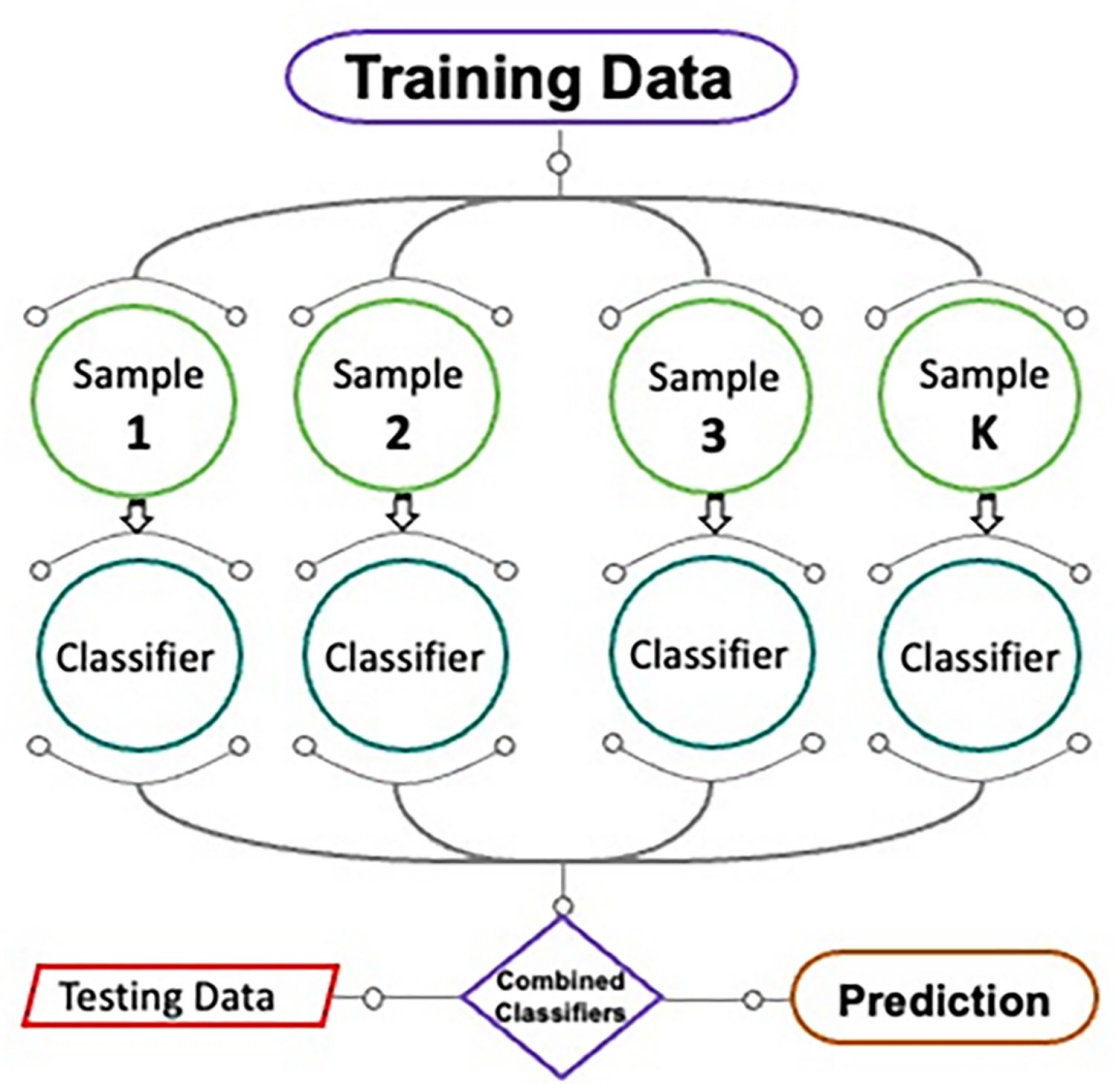
Sequence of BR technique [70].

A schematic diagram of the BR method is shown in Fig 5. A similar ensemble technique underlies the adjustment of the prediction model caused by the inclusion of additional training data. Data from the primary set is substituted in the irregular sampling procedure. Each new training data sample using replacement sampling may contain identical observations. After bagging, the chance of occurrence for each component in the new data sample is the same. The projecting force has no effect on the quantity of the training dataset. In addition, by improving the estimation of the required outcome, the divergence may be significantly reduced and training further models using these data samples. The mean of all model forecasts is utilized for this ensemble. The mean of the estimations from many models can be used as an estimate in regression [69]. To fine-tune the bagging strategy using SVM and find the optimal output-generating value, twenty submodels are used.

#### 2.2.3. Validation methods for prediction models

**Fig 6:**
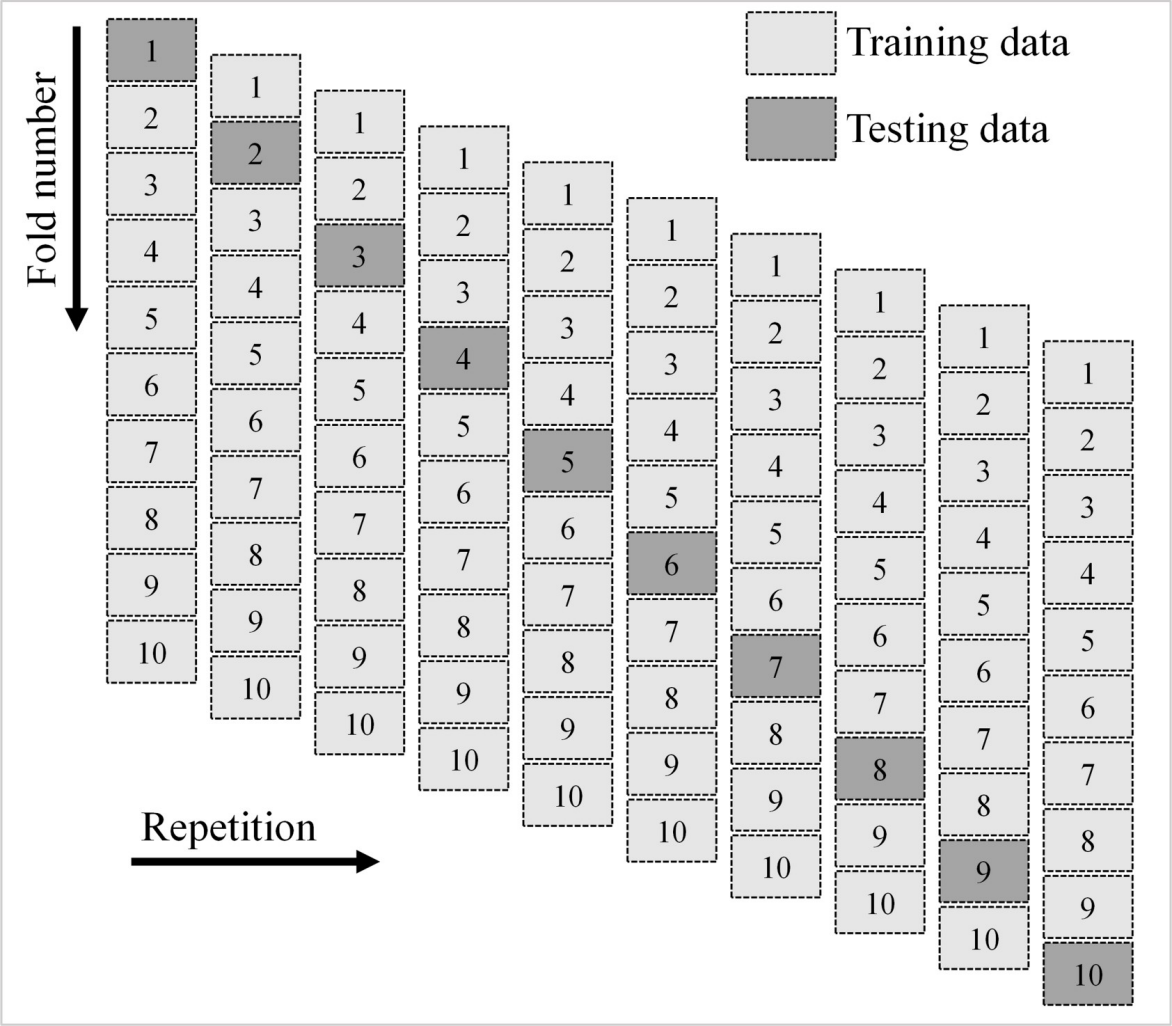
Schematic diagram of the k-fold validation technique.

The used ML algorithms were verified using k-fold and statistical methods. For technique evaluation, the k-fold approach of randomly dividing data into 10 groups is commonly used [71]. As can be seen in Fig 6, ML models are trained using nine classes and validated using a single class. If the errors are smaller and the R^2^ value is higher, then the ML technique is more accurate. Additionally, the process needs to be repeated ten times for the desired result to be realized. The incredibly high precision of the model is largely due to this effort. Errors assessment was also used to statistically compare the precision of different ML methods (MAE, RMSE, and MEPE). Eqs (1)–(3), derived from previous studies [52,72], were used to statistically evaluate the projection accuracy of the ML methods.

$$MAE=\frac{1}{n}\sum_{i=1}^{n}\left|P_{i}-T_{i}\right|,$$ (1)

$$RMSE=\sqrt{\sum \frac{{\left(P_{i}-T_{i}\right)}^{2}}{n}},$$ (2)

$$MAPE=\frac{100\%}{n}\sum_{i=1}^{n}\frac{\left|P_{i}-T_{i}\right|}{T_{i}},$$ (3)

where *n* = dataset size, *P*_*i*_ = projected outcomes, and *T*_*i*_ = actual outcomes.

## 3. Results and analysis

### 3.1. Experimental results

**Fig 7:**
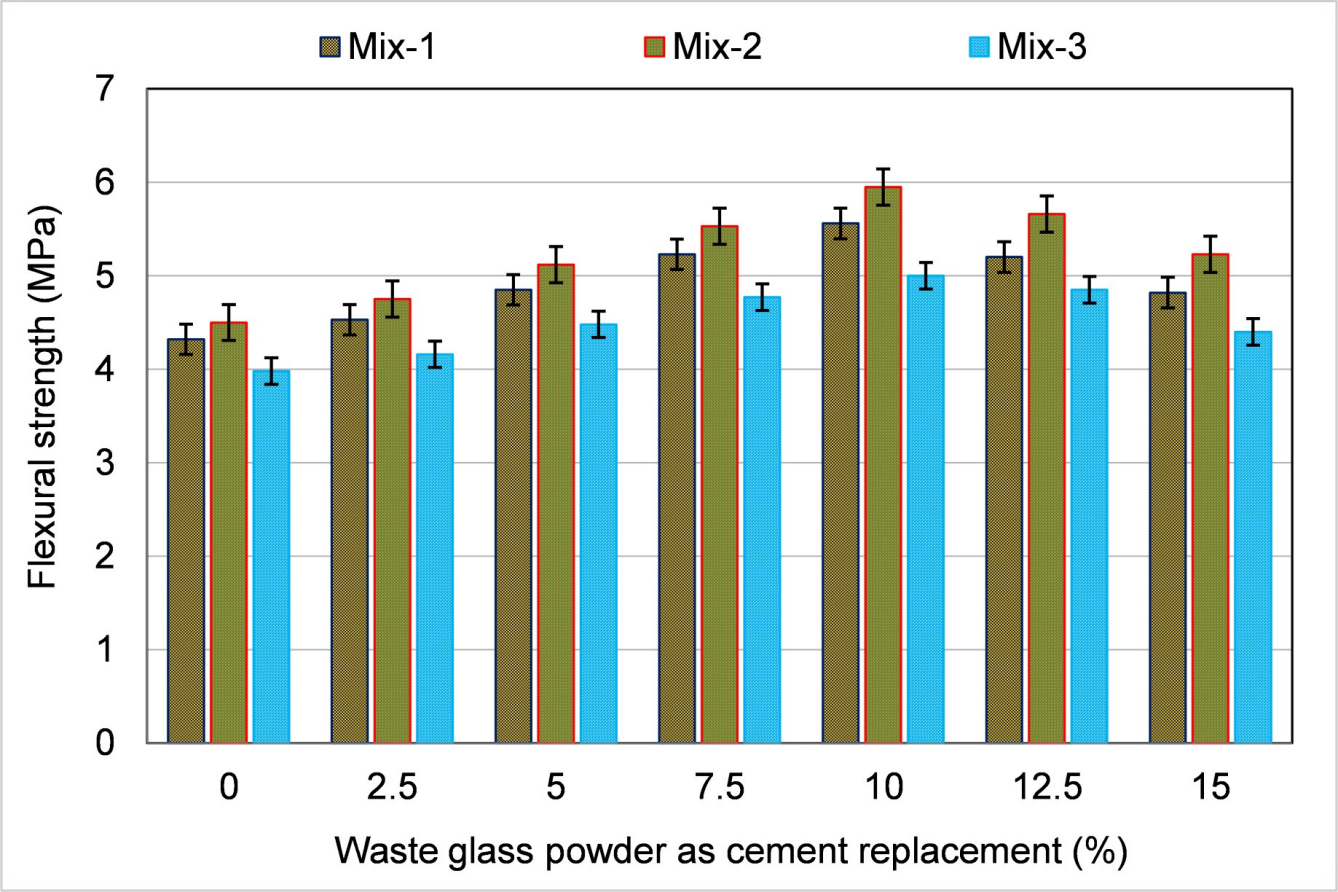
Influence of waste glass powder as cement substitute on FS of specimens.

Fig 7 displays the FS for CC specimens WGP as a partial replacement for cement. As anticipated, the addition of WGP resulted in the enhancement of FS. The FS increased with a rising WGP percentage of up to 10% in all mixes, whereas the further increase in WGP proportion caused a decrease in FS. However, the FS of the samples with 12.5% and 15% WGP as cement replacement were also greater than the FS of the control sample with 0% WGP. For instance, in Mix-1 samples, the FS of CCs enhanced by 4.9%, 12.3%, 21.1%, 28.7%, 20.4%, and 11.6% compared to the control specimen (0% WGP) with 2.5, 5, 7.5, 10, 12.5, and 15% WGP ratio, respectively. Comparable findings were also noticed in other mixes (2 and 3), and the highest FS was accomplished at 10% WGP proportion as cement substitute, which was nearly 32% and 26% higher than the reference specimen in Mixes-2 and 3 specimens, respectively. The probable causes are the filling effect and the pozzolanic nature of WGP [30]. The filling effect lowers the void ratio, causing a dense and compact structure. The higher content of SiO_2_ in the glass’s composition [73] interacts with Ca(OH)_2_ in the mix to form improved calcium-silicate-hydrate (C-S-H) gel, improving the characteristics of CCs [74,75]. At greater proportions of WGP (12.5 and 15%), the FS declined due to the excess quantity of WGP incorporated into mixes than needed for the pozzolanic activity [30] and cement dilution. Therefore, the application of WGP up to 10% cement substitute is advantageous for attaining the highest strength.

**Fig 8:**
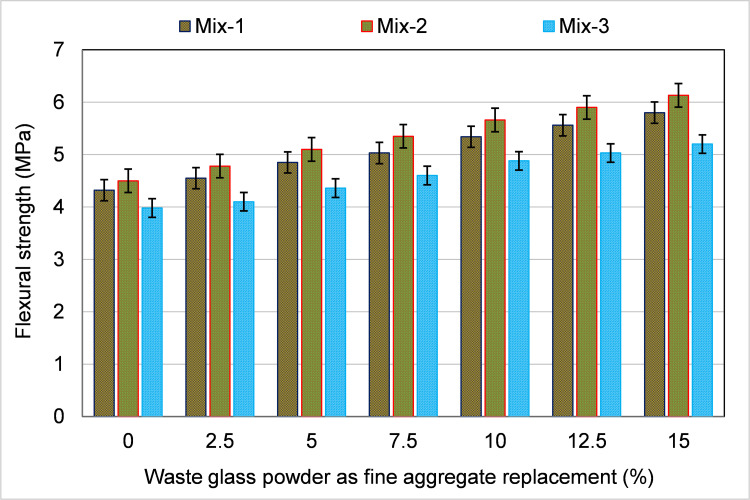
Influence of waste glass powder as a fine aggregate substitute on FS of specimens.

The FS of the samples incorporating WGP as a fine aggregate substitute is displayed in Fig 8. The incorporation of WGP increased the FS at all contents in each mix, and at higher contents, the FS improvement was more. For instance, the FS of samples in Mix-1 increased up to 34.3% at 15% WGP content relative to the control mix (0% WGP). A likewise improving trend in the FS was also noticed in Mixes-2 and 3 specimens. The improvement in FS was found to be about 36% and 31% in Mixes-2 and 3 specimens, respectively, at a 15% content as a fine aggregate substitute. The key cause for the rise in FS may be due to the enhanced grain packing, as WGP grains were finer than fine aggregate particles [76]. Additionally, the incorporation of WGP in CCs also developed a pozzolanic reaction, which improved hydration products like C-S-H gel and ultimately enhanced the load-carrying capacity of the sample [30]. Therefore, WGP may be used as fine aggregate up to a 15% replacement ratio for achieving increased strength.

### 3.2. Prediction models

#### 3.2.1. Results of the SVM model

**Fig 9:**
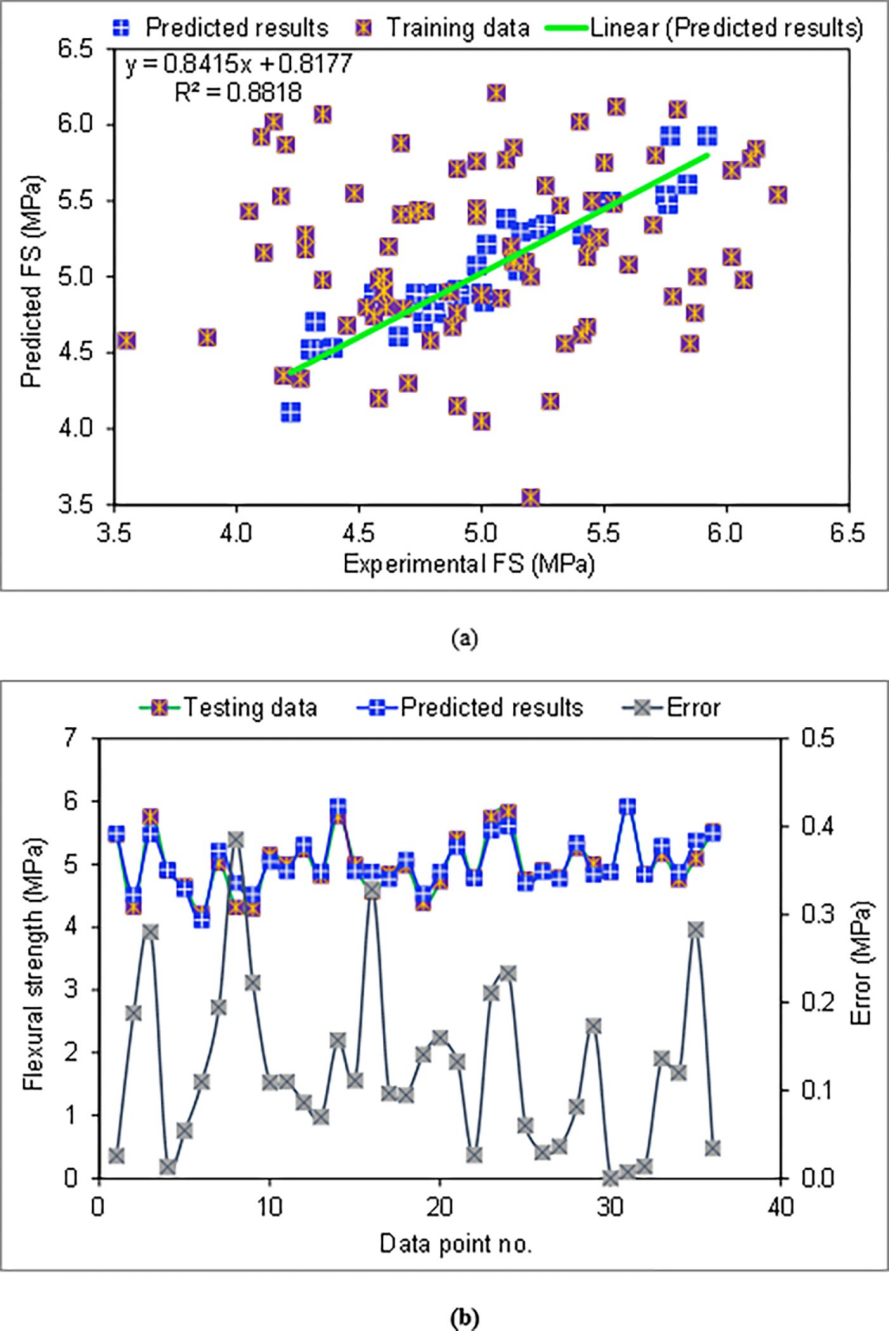
SVM model: (a) Correlation among actual and estimated FS; (b) Dispersion of actual, estimated and error results.

The results of the SVM method applied to the estimation of the FS of CCs with varying amounts of WGP are displayed in Fig 9. Fig 9(A) depicts the correlation amongst actual FS and estimated FS. As a result of using the SVM method, accurate results were obtained, with little distinction among experimental and predicted values. The experimental and projected findings correspond well, and the R^**2**^ result of 0.88 indicated that the SVM technique for estimating the FS of CCs is adequate. The variation in experimental, anticipated, and divergent values (errors) for the SVM algorithm is shown in Fig 9(B). The distribution of the errors was 0.13 MPa on average, while the maximum was 0.39 MPa. The assessment of the error value distribution found that 44.4% of the values were less than 0.1 MPa, 36.1% were in the range of 0.1 to 0.2 MPa, and 19.4% were higher than 0.2 MPa. The SVM approach correctly predicted the FS of CCs incorporating WGP, as validated by the distribution of errors.

#### 3.2.2 Results of the BR model

**Fig 10:**
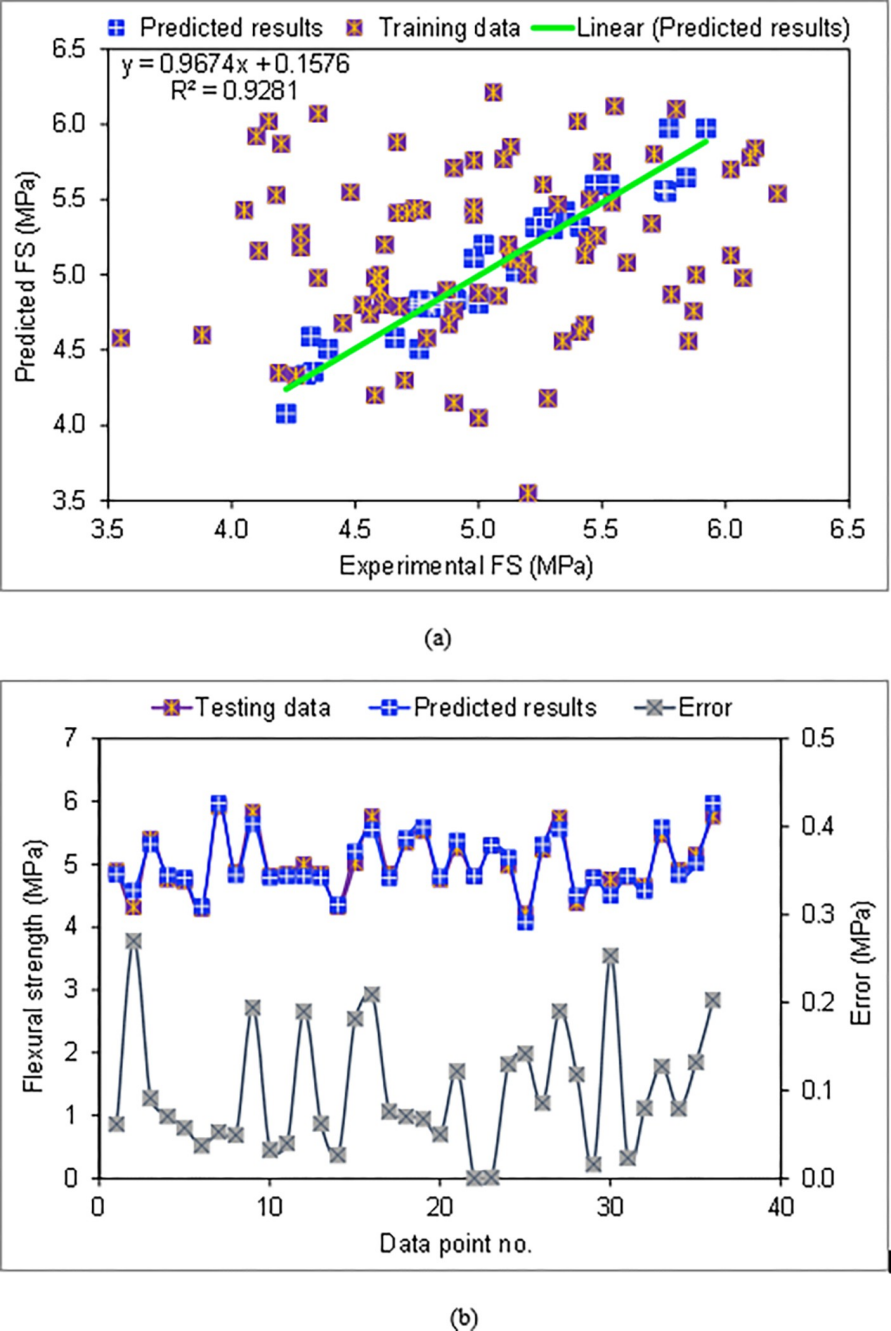
BR model: (a) Correlation among actual and estimated FS; (b) Dispersion of actual, estimated and error results.

Results of the BR technique applied to predict the FS of the CCs incorporating WGP are presented in Fig 10. Fig 10(A) illustrates the correlation amongst actual and estimated FS. As matched to the SVM technique utilized in the present study, the BR method yielded more exact outcomes and showed the least discrepancy amongst actual and estimated FS. The BR model has a higher R^**2**^ of 0.93, implying greater accuracy. The BR method’s error distribution is shown in Fig 10(B). It was revealed that the error ranged from 0.07 to 0.27 MPa, with a mean of 0.10 MPa. Analyzing the error dispersal revealed that 61.1% were lower than 0.1 MPa, 27.8% were amongst 0.1 and 0.2 MPa, and 11.1% were higher than 0.2 MPa. Therefore, the error dispersal demonstrated that the BR approach is more exact than the SVM that was employed. By employing SVM to fine-tune the bagging algorithm over 20 separate submodels, the BR approach is more exact than SVM since it is an induvial algorithm [77].

### 3.3. Validation of ML models

**Fig 11:**
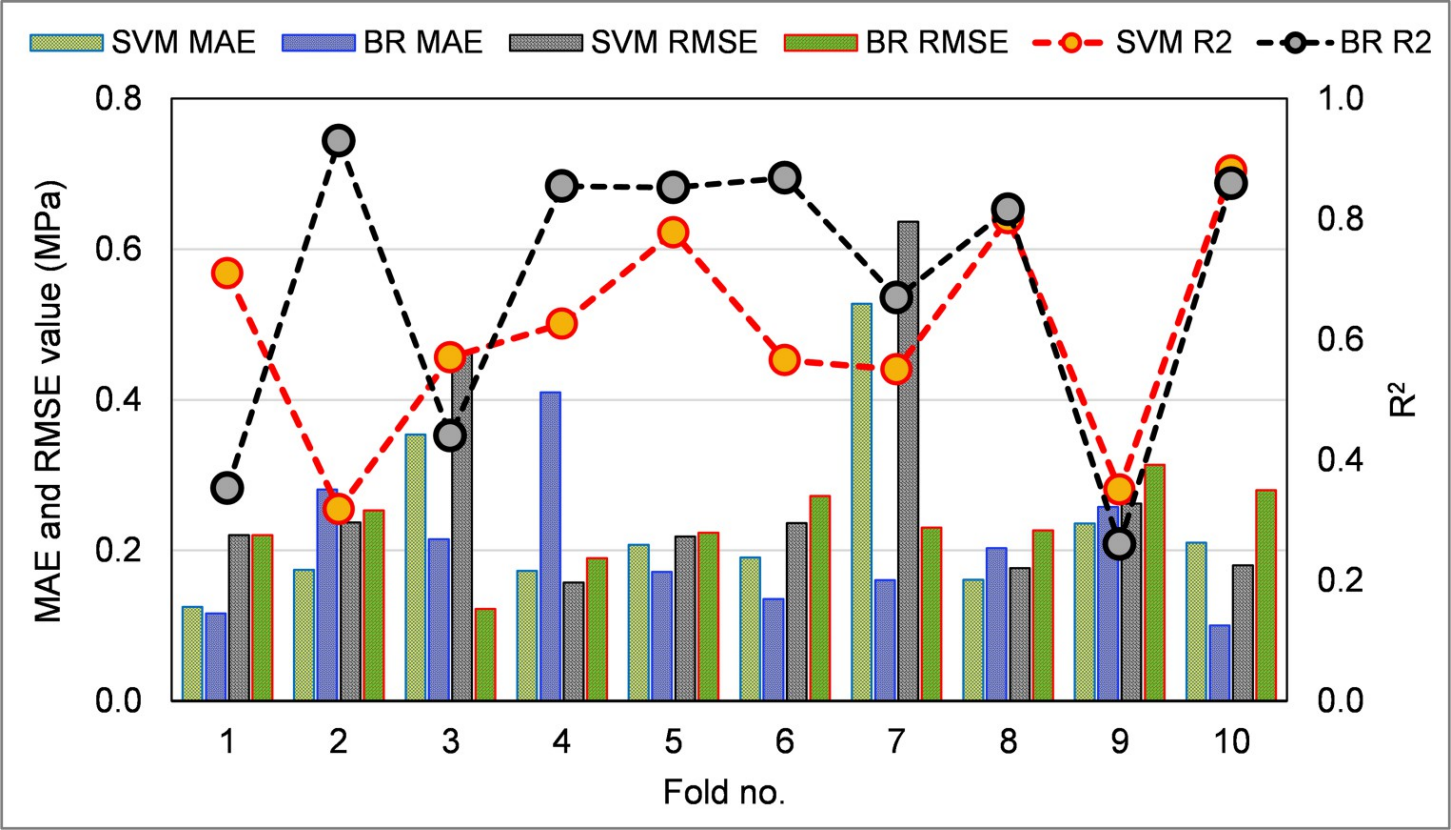
Outcomes of k-fold evaluation.

Table 2 displays the MAPE, RMSE, and MAE results from statistical tests performed using Eqs (1)–(3) above. The MAE for SVM and BR were found to be 0.125 MPa and 0.100 MPa. Results showed that the MAPE for SVM was 2.5%, and for BR, it was 2.0%. The RMSE for SVM was determined to be 0.157 MPa, whereas the RMSE for BR was 0.122 MPa. Furthermore, these evaluations demonstrated that the BR model, with its reduced error rate, is more accurate than the SVM. Table 3 displays the results of computing R^2^, MAE, and RMSE for the purpose of validating the models using the k-fold approach. Fig 11 was made so that the results of k-fold studies using both ML techniques could be compared. The SVM method has an MAE averaging 0.24 MPa, with a range of 0.13 MPa to 0.53 MPa. The MAE for the BR model was 0.10 MPa to 0.41 MPa, on average. In the same way, the RMSE for the SVM model was 0.28 MPa, whereas the RMSE for the BR model was 0.23 MPa on average. The BR model’s R^2^ was 0.69, and the SVM model was 0.61 on average. According to the k-fold test, the BR model with the smaller deviations and higher R^2^ is the most effective in forecasting the FS of CCs incorporating WGP.

**Table 2:** Statistical checks for the employed ML methods.

ML approach	MAE (MPa)	MAPE (%)	RMSE (MPa)
SVM	0.125	2.50	0.157
BR	0.100	2.00	0.122

**Table 3:** Results of k-fold evaluation for the employed ML models.

K-fold	SVM			BR		
	MAE	RMSE	R^2^	MAE	RMSE	R^2^
1	0.13	0.22	0.71	0.12	0.22	0.35
2	0.17	0.24	0.32	0.28	0.25	0.93
3	0.35	0.46	0.57	0.21	0.12	0.44
4	0.17	0.16	0.63	0.41	0.19	0.85
5	0.21	0.22	0.78	0.17	0.22	0.85
6	0.19	0.24	0.57	0.14	0.27	0.87
7	0.53	0.64	0.55	0.16	0.23	0.67
8	0.16	0.18	0.80	0.20	0.23	0.82
9	0.24	0.26	0.35	0.26	0.31	0.26
10	0.21	0.18	0.88	0.10	0.28	0.86

## 4. Discussions

The FS of CCs with WGP as a partial substitution for cement and fine aggregate was evaluated in this work using both experimental and modeling methodologies. There is an excessive volume of WG produced globally, and most of it is disposed of in landfills, where it poses health and environmental risks to people and the atmosphere [30]. Additionally, CCs are the most often used construction materials, and their increasing demand leads to the depletion of natural raw materials and the release of CO_2_. The use of WG in CCs as a cement and fine aggregate substitute has the potential to be an environmentally beneficial technique. Thus, avoiding waste, conserving natural raw materials, and reducing CO_2_ emissions are some of the aspects due to which the use of WG in CCs will lessen their negative effects on the environment. Therefore, this research aimed to expand our understanding of WGP’s role in CCs by integrating experimental and ML-based modeling techniques. Different percentages of WGP were used to substitute cement and fine aggregate in the casting of CC samples (0–15%) with a 2.5% increment. The experimental findings showed that adding WGP enhanced FS. As a cement substitute, a WGP concentration of 10% yielded the highest FS, up to 32% more relative to the reference mix. Amongst the potential reasons are the filling effect and the pozzolanic reactivity of WGP. The matrix is dense and compact due to the filling effect, which decreases the void ratio. A higher percentage of SiO_2_ in glass’s composition combines with Ca(OH)_2_ in the matrix to develop improved C-S-H gel, which increases the material’s characteristics [74,75]. Since more WGP was used than required for the pozzolanic process and cement was diluted, the FS decreased with higher WGP concentrations (12.5% and 15%) as a cement replacement [30]. To get the highest possible strength, it is recommended to utilize WGP as a cement replacement at a concentration of up to 10%. When WGP was used to replace fine aggregate in CCs, the FS was up to 36% greater than in the control mix at 15% replacement. WGP being finer than finer aggregate may have improved particle packing, which in turn led to a higher FS [76]. WGP was also shown to enhance hydration products like C-S-H gel and the material’s FS by a pozzolanic interaction with cement [30]. Therefore, in order to achieve optimum strength, WGP can be used as a fine aggregate substitute at a percentage of up to 15%. Although WGP shows potential, more study is needed to determine its value at higher replacement rates.

The ML models were executed on the organized experimental data. When attempting to estimate the FS of WGP-based CCs, both a single ML technique (SVM) and an ensemble technique (BR) were utilized. Both approaches were evaluated for their exactness in predicting outcomes. The R^2^ for the BR model was 0.93, which was higher than the R^2^ for the SVM (0.88). Lower error values were found for the BR model when compared with SVM, confirming its superior accuracy. Nevertheless, the SVM model’s prediction was also accurate and in agreement with the experimental data. It has been established via previous research that the BR approach is more accurate compared to the individual ML methods in predicting the strength characteristics of various materials [77–80]. For example, Alsharari et al. [77] forecasted the compressive strength of cement mortar using BR and SVM ML techniques. According to reports, the performance of BR methods was found to be superior to the SVM in estimating the compressive strength of cement mortar. The success of an ML strategy is highly reliant on the input variables and data sample used to execute methods [52], making it difficult to define and propose the optimal ML method for forecasting outcomes in different research areas. When comparing single ML methods to ensemble ML approaches, it’s important to note that the ensemble ML algorithms frequently use the weak learner by building submodels that are trained on the data sample and adjusted to increase the R^2^ value. As a result, the ensemble ML models produced more precise results than the individual. The construction industry may profit from this type of research because it will facilitate the development of efficient methods for assessing material attributes quickly and cheaply.

## 5. Conclusions

The purpose of this study was to study the flexural strength (FS) of cementitious composites (CCs) incorporating waste glass powder (WGP) by using both experimental and ML methodologies. The FS of samples was calculated via experiments, and the resulting data sample was utilized to build ML prediction models. Support vector machine (SVM) and bagging regressor (BR) are two machine learning (ML) techniques that were used to make predictions about the FS. The findings of this study are:

- The experiments’ results showed that adding WGP might improve the FS of CCs. When used as a cement substitute, WGP increased FS by up to 32% when used at a 10% concentration. Both the filling effect and the pozzolanic feature of WGP have been anticipated as potential explanations.
- At 15% replacement, FS was 36% greater than the reference sample when WGP was utilized as a fine aggregate substitute in CCs. The pozzolanic nature of the glass and the fact that WGP was finer than finer aggregate both have the potential to explain the improved FS.
- Based on the results of the ML models, it was discovered that the SVM model was accurate with an R^2^ of 0.88, while the BR technique was more precise with an R^2^ of 0.93 in predicting the FS of CCs incorporating WGP.
- Statistical and k-fold evaluations verified the effectiveness of the used model. Improved R^2^ and lower error rates were indicators of how well ML models performed. SVM and BR models were found to have MAPEs of 2.5% and 2.0%, respectively. The MAPE values demonstrated that the BR model provided the most accurate forecasts of FS.
- Sustainable development is aided by the use of recycled glass in building materials because it prevents the waste glass from being dumped, conserves natural raw materials, generates cost-effective materials, and reduces CO_2_ emissions.
- The construction industry may benefit from the adoption of cutting-edge techniques, such as ML because it will speed up the development of more cost-effective and time-efficient ways for determining material properties.

This study was limited to investigating the effect of using WSP on the flexural strength of the CCs. However, for the potential engineering applications of the material, future research is directed to fully explore the other aspects as well, such as compressive and split tensile strength and durability. Additionally, the strength of CCs can also be affected by other factors, such as the water-to-binder ratio, curing conditions, the quality of raw materials, and environmental effects (temperature and humidity), so it will be necessary to develop a dataset containing these input variables in the future for ML modeling.
